# Genetic studies on iron and zinc concentrations in common bean (*Phaseolus vulgaris* L.) in Ghana

**DOI:** 10.1016/j.heliyon.2023.e17303

**Published:** 2023-06-15

**Authors:** Maxwell Lamptey, Hans Adu-Dapaah, Francis Osei Amoako-Andoh, Louis Butare, Kwabena Asare Bediako, Richard Adu Amoah, Isaac Tawiah, Stephen Yeboah, James Yaw Asibuo

**Affiliations:** a Council for Scientific and Industrial Research (CSIR)-Crops Research Institute, Kumasi, Ghana; b Alliance of Bioversity International and International Center for Tropical Agriculture (ABC) Africa Hub, Italy; c Cocoa Research Institute of Ghana, P.O. Box 8, New Tafo-Akim, Ghana; d Council for Scientific and Industrial Research (CSIR)-Plant Genetic Resources Research Institute, Bunso, Ghana; e AfricaRice M'bé Research Station, 01 BP 2551, Bouaké, Cote d’Ivoire; f Department of Plant Resources Development, CSIR College of Science and Technology, Fumesua-Kumasi, Ghana

**Keywords:** *Phaseolus vulgaris*, Biofortification, Heritability, Generation mean analysis, Zinc, Iron

## Abstract

Iron and zinc deficiencies cause high health risk to young children and expectant mothers in sub Saharan Africa. The development of biofortified common bean (*Phaseolus vulgaris* L.) varieties could address the acute micronutrient deficiencies with associated improvement in the nutrition and health of women, children and adults. The objective of this study was to determine the mode of gene action and genetic advance in iron and zinc levels in common bean. Field experiment was carried out using six generations of two populations made of crosses between pairs of low iron, low zinc and high iron, moderate zinc genotypes (Cal 96 ˣ RWR 2154; MCR-ISD-672 ˣ RWR 2154). Each generation (P_1_, P_2_, F_1_, F_2_, BC_1_P_1_ and BC_1_P_2_) was evaluated on the field in a randomized complete block design with three replications. Generation mean analysis were performed for each trait measured in each of the crosses while iron and zinc levels were quantified by x-ray fluorescence. The study showed that both additive and non-additive gene effects were important in determining the expression of high iron and zinc levels. Iron concentration in the common bean seeds ranged from 60.68 to 101.66 ppm while zinc levels ranged from 25.87 to 34.04 ppm. Broad sense heritability estimates of iron and zinc were high in the two crosses (62–82% for Fe and 60–74% for Zn) while narrow sense heritability ranged from low to high (53–75% for Fe and 21–46% for Zn). Heritability and genetic gain were used as selection criteria for iron and zinc, and it was concluded that doing so would be beneficial for future improvement.

## 1 Introduction

Common bean (*Phaseolus vulgaris* L.) known as kidney bean, dry bean, navy bean, snap bean, haricot bean or French bean is considered as one of the most important protein and mineral source in developing countries [[[1], [2], [3]]. Seed iron concentration in bean varieties varied from 40 to 120 ppm and zinc concentration from 20 to 52 ppm [4]. Given the importance of beans as a source of inexpensive protein and minerals, the crop plays an important role in reducing poverty and improving food security [5]. Although, world production trend of common bean is scanty, it is estimated that 12 million tons is produced annually. The production of common bean in Africa is estimated to be around 5.9 million tons of which is mainly centered in the Eastern and Central regions [6]. In Ghana, the crop was recently introduced from Uganda following evaluation of introduced lines, and official release and commercialization of adapted varieties in 2016.

Over 3 billion people worldwide suffer from malnutrition due to iron and zinc deficiencies [[7], [8], [9], [10]]. Iron deficiency in children and adolescence adversely affect physical growth, mental development and learning capacity. In adults, iron deficiency causes anaemia which reduces the capacity to do physical labour. Iron deficiency also increases the risk of women dying during delivery or in the post-delivery period. Zinc deficiency has been linked to stunting in children [7,8,11]. In Ghana, sixty-six percent (66%) of children between 6 and 59 months and forty-two (42%) of women are anaemic [12]. Research conducted in Ghana shows that micronutrient deficiency is a major contributor to child morbidity and mortality. In women, anaemia has resulted in complications of pregnancy among them being poor foetal development and death [12]. This situation has created a serious problem in Ghana, hence the need to improve micronutrient content in common bean cultivars would contribute in addressing malnutrition. The development and adoption of biofortified beans will go a long way to address the acute micronutrient deficiencies with concomitant improvement in the nutrition and health for women, children and men in Ghana.

The magnitude of genetic variability within breeding populations has important bearing on crop improvement [3]. Generation mean analysis provides estimates of epistatic effects information on the relative importance of average effects of the genes (additive effects), dominance deviations and effects due to non-allelic genetic interactions to guide breeding and selection for quantitative traits [13]. The success of genetic improvement depends on sufficient genetic variability and heritability of the trait under consideration. Therefore, the objectives of the current study were to determine the mode of gene action, the extent of genetic variability, heritability and genetic gain for iron and zinc contents of the two common bean crosses.

## 2 Materials and methods

### 2.1 Experimental materials

**Fig. 1:**
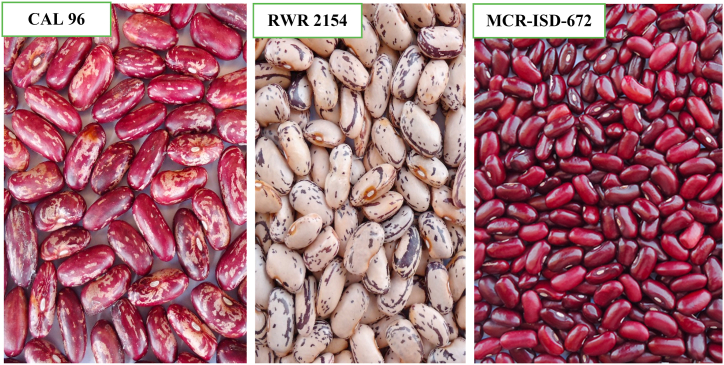
Seeds of parents used in the study of genetic variability and heritability.

Three (3) genotypes (Fig. 1 and Table 1) received from the Alliance Bioversity International and International Center for Tropical Agriculture (CIAT)-Uganda were used as parental materials. MCR-ISD-672 is small seeded, high yielding but low in iron and zinc. RWR 2154 is large seeded, early maturing, high but low in iron and zinc contents respectively. RWR 2154 is a released material in Rwanda as a bushy plant type but climbs profusely in Ghana. The low Fe/Zn parents included, CAL 96, a red mottled bean developed and released in Uganda. It is highly susceptible to all major diseases and is used as a local disease and high yield check [14]. Two single crosses were made using the three parents; cross 1 (CAL 96 ˣ RWR 2154) and cross 2 (MCR-ISD-672 ˣ RWR 2154). The F_1_ seeds generated from the parents were planted to generate F_2_ population through selfing and the backcross populations (BC_1_P_1_ and BC_1_P_2_) were simultaneously generated through crossing F_1_ hybrids back to their respective parents (P_1_ and P_2_). The experimental materials consisted of six generations for each cross include parents (P_1_ and P_2_), the first and second filial generations (F_1_ and F_2_) and backcrosses with the donor or recurrent parent (BC_1_P_1_ and BC_1_P_2_) (Table 2).

**Table 1:** Unique traits of parental materials.

Line	Fe (ppm)	Zn (ppm)	Growth type	Seed colour	seed size^a^
CAL 96	Low (56)	Low (30)	Bush bean	Red mottled	Large
MCR-ISD-672	Low (61)	Low (26)	Climber	Red	Small
RWR 2154	High (85)	Moderate (34)	Bush bean	Sugar	Large

a Large (>40 g/100 seeds); medium (26–39 g/100 seeds); small (<25 g/100 seeds).

**Table 2:** List of parents and crosses produced.

Generation	(Population 1)	(Population 2)
P_1_	MCR-ISD-672	CAL 96
P_2_	RWR 2154	RWR 2154
F_1_	MCR-ISD-672 ˣ RWR 2154	CAL 96 ˣ RWR 2154
F_2_	MCR-ISD-672 ˣ RWR 2154 (selfed)	CAL 96 ˣ RWR 2154 (selfed)
BC_1_P_1_	(MCR-ISD-672 ˣ RWR 2154) ˣ MCR-ISD-672	(CAL 96 ˣ RWR 2154) ˣ CAL 96
BC_1_P_2_	(MCR-ISD-672 ˣ RWR 2154) ˣ RWR 2154	(CAL 96 ˣ RWR 2154) ˣ RWR 2154

### 2.2 Site description

Crosses to generate six basic generations and field experiment were carried out at Fumesua Crops Research Institute (CRI) of the Council for Scientific and Industrial Research of Ghana (CSIR) in the Ashanti Region of Ghana (01° 36°W; 06° 43°N) from July 2017 to January 2018 and during the major planting season from May to August 2018, respectively. Fumesua is in the Semi-deciduous forest zone with elevation of 186 m above sea level. Fumesua has a bimodal rainfall distribution with an average total annual rainfall of about 1,727.2 mm. The major rainfall season is from March to July while the minor rainfall season is from August to November. The mean annual relative humidity is 95% in the morning and 61% at noon. The mean minimum and maximum temperatures are 21 °C and 31 °C, respectively. The soil at the experimental site belongs to the Asuansi series and is classified as Ferric Acrisol.

### 2.3 Soil analysis

Soil at the experimental site was analyzed at the Department of Crops and Soil Sciences of the Kwame Nkrumah University of Science and Technology (KNUST) for Iron (Fe), Zinc (Zn), acidity (pH), organic matter (OM), organic carbon (OC), Nitrogen (N), Phosphorus (P), Potassium (K), Calcium (Ca), Magnesium (Mg), Copper (Cu) and Manganese (Mn).

### 2.4 Experimental design

Randomized complete block design (RCBD) with three replications was used for the field evaluation. Six treatments (P_1_, P_2_, F_1_, F_2_, BC_1_P_1_ and BC_1_P_2_) for each cross were used. Each replication had 14 plants in two rows for each of the parents (P_1_ and P_2_) and F_1_ populations, 28 plants in four rows for backcross populations, and 70 plants in ten rows for the F_2_ populations. Plant spacing of 50 × 30 cm, 2 m plot length with 1 m between plots and 2 m between replications were used in the experimental design. Seeds were planted at a density of one per hole.

Standard agronomic procedures were followed for proper plant development. Triple Super Phosphate (TSP 46% P_2_O_5_) was applied as side placement fertilization for each plant at a recommended rate of 100 kg P_2_O_5_/ha. The trial was monitored for insect pest control with Lambda Super 2.5 EC (0.4–0.8 L/ha) and Dimethoate 40 EC (0.2–0.3 L/ha) at pre-flowering and post-flowering stages, respectively.

### 2.5 Iron and zinc quantification

Matured pods were allowed to dry before harvesting and threshed by hand to avoid soil contamination. Clean, dirt free and dry pods were harvested before the main harvest. The harvested pods were hand threshing and seeds were subsequently dried to a moisture content of 12–14%, out of which 40 seeds were counted from each plant. The seeds were cleaned with dry cotton cloth, packed and shipped to Rwanda Agriculture Board (RAB) Rubona Research Station for rapid screening of iron (Fe) and zinc (Zn) content in common bean seeds. For quantification of iron and zinc, X-ray fluorescence (XRF) method was used for analyzing and determining Fe and Zn in the grains [15,16]. Briefly, the samples were thoroughly washed and cleaned with distilled water and clean cloth to avoid aluminium contamination. The samples were then oven dried at 60 °C for 12 h and then ground using Sunbeam Conical Burr Mill EM0480 Grinder. The ground samples were transferred into small sample cups on a tray positioned in the machine's tray. Iron and zinc content in the seeds was determined using X-Ray Fluorescence Spectrometer (XRF) by analyzing each sample for 100 s with spinning of sample cup to determine iron and zinc contents and record intensities of X-rays emitted from the generator. The screen tray rotates to place the samples being measured at the top and the results of the analysis displayed automatically when all the samples on the tray have been measured.

### 2.6 Data analyses

Analysis of variance (ANOVA) and mean separation of the six generations of each cross combination were done using GenStat version 12 software [17]. Treatment means were separated using Tukeys Honesty Significant Difference (HSD) at 5% level of significance test.

### 2.7 Generation mean analysis

Generation mean analysis was carried out with R package for determination of gene action for high iron and zinc. The observed means of the six generations and their standard errors were used to estimate the mid-parent (m), additive (a) and dominance (d) gene effects using the joint scaling test of [18]. The adequacy of the simple additive-dominance model (mean, additive and dominance effects) was determined by χ^2^ test. Individual scaling tests (A, B, C and D) of [19,20] were employed to test their fitness to the additive-dominance model. The significance of any of these four scales indicated the presence of epistasis. In case of the inadequacy of the three-parameter genetic model and significance of the scaling tests, six-parameter genetic model suggested by Ref. [21] were used to estimate various genetic components.

Where the simple model proved to be inadequate, epistasis additive x additive [aa], additive x dominance [ad] and dominance x dominance [dd] were added to the model, as proposed by Ref. [21]. The significance of genetic parameters (m, [a], [aa], [ad] and [dd]) were tested using *t*-test.

Broad-sense and narrow-sense heritability were estimated by Ref. [22] as follows:

(1) $$H^{2}=\left[{VF}_{2}\;–\;\left({VP}_{1}+{VP}_{2}+{VF}_{1}\right)/3\right]/{VF}_{2}$$

(2) $$h^{2}=\left[2{VF}_{2}\;–\;\left({VBC}_{1}+{VBC}_{2}\right)\right]/{VF}_{2}$$

The components of variation in six populations were calculated by the formulae of F_2_ variance were obtained by the following formula of [18] as:

(3) $$E\;{(\sigma }_{E}^{2})=1/3\left({VP}_{1}+{VP}_{2}+{VF}_{1}\right)$$

(4) $$D=4{VF}_{2}\;–\;2\left({VBC}_{1}+{VBC}_{2}\right)$$

(5) $$H=4\left({VF}_{2}\;–\;1/2VD\;–\;VE\right)$$

(6) $$F={VBC}_{1}\;–\;{VBC}_{2}$$

(7) $$\sigma_{P}^{2}={VF}_{2}$$

(8) $$\sigma_{g}^{2}=\left(\sigma_{P}^{2}-\sigma_{E}^{2}\;\right)$$

Where:

D – additive genetic variance
H – dominance Variance
E − environmental component of variance
VF_2_ = variance of second filial generation, VBC_1_ = variance of the first backcross to the first parent, VBC_2_ = variance of the first backcross to the second parent, $\sigma_{P}^{2}$ = phenotypic variance, $\sigma_{G}^{2}$ = genotypic variance, $h^{2}$ = narrow sense heritability, $H^{2}$ = broad sense heritability

Estimates of heritability were classified according to low (0–30%), moderate (31–60%) and high (>60%) [23].

### 2.8 Genetic advance (GA) and genetic advance as percentage of mean (GAM)

Genetic advance (GA) was calculated according to Ref. [24] with selection intensity of i = 5% for all the characters as follows:

(9) $$GA=i.\;H^{2}.\sqrt{{VF}_{2}}$$

(10) $$GAM=\frac{GA}{\bar{X}}\;x\;100$$

Where,

$H^{2}$ = broad-sense heritability, i = selection intensity at 5% (i = 2.06) [24]. categorized genetic advance as percent of mean (GAM) as low (<10%), medium (10–20%) and high (> 20%).

## 3 Results and discussion

### 3.1 Analysis of variance

The analysis of variance showed highly significant (p < 0.001) variation among the generations in iron and zinc for crosses 1 and 2 (Table 3). This indicates that selection for improvement of iron and zinc would be effective. Although there was significant genetic diversity among the populations, lower coefficient of variation (CV) recorded showed the reliability of the results from the XRF method of quantification.

**Table 3:** Mean squares for Iron (ppm) and Zinc (ppm) in two crosses of common bean.

Source of variation	CAL 96 ˣ RWR 2154			MCR-ISD-672 ˣ RWR 2154	
	DF	Fe	Zn	Fe	Zn
REP	2	31.03	8.35	40.93	6.62
GEN	5	1854.55***	84.76***	1323.49***	78.41***
RESIDUAL	70	123.30	16.37	40.93	12.84
CV (%)		12.55	12.92	15.12	11.53

GEN = generations, REP = replications, CV = coefficient of variation, ***significant at 0.001, **significant at 0.01, *significant at 0.05.

### 3.2 Treatment mean comparisons

Mean values and standard errors (SE) for iron and zinc for the two crosses are presented in Table 4. The mean values of iron were higher in progenies of all the two crosses than zinc. This may be due to high variability observed in iron content than zinc content. Parents in two of the crosses (CAL 96 ˣ RWR 2154 and MCR-ISD-672 ˣ RWR 2154) showed significant differences (P ≤ 0.05) in iron and zinc, indicating that there were significant genetic variations among the parents for these micronutrients. The results also imply that generation mean analysis could be carried out for the two crosses. Contrasting parents are prerequisite to accurate estimation of the number of genes and genetic parameters for the traits [25] The F_1_ hybrids in the cross CAL 96 ˣ RWR 2154 recorded significantly (P ≤ 0.05) higher iron content (101.66 ppm) than its parents. Further, the mean zinc concentration of F_1_ hybrids was significantly different from the parent with lower zinc content (P_1_) and not different from the parent with higher zinc content (P_2_). The mean iron and zinc contents of hybrids in cross MCR-ISD-672 ˣ RWR 2154 were significantly different from P_1_ and not different from P_2_. The results suggest dominance in the direction of the parent with higher iron and zinc contents (RWR 2154). Thus, genes for high iron and zinc contents were transmitted from the male parent (RWR 2154) to its offspring.

**Table 4:** Mean and standard error of iron and zinc concentration in common beans measured by x-ray fluorescence (XRF) in parents (P_1_ and P_2_) and their F_1_, F_2_, BC_1_P_1_ (F_1_ ˣ P_1_) and BC_1_P_2_ (F_1_ ˣ P_2_).

Generation	Cross 1		Cross 2	
	Cal 96ˣ RWR 2154		MCR-ISD-672 ˣ RWR 2154	
	Fe	Zn	Fe	Zn
P_1_	61.61 ± 3.70^a1^	25.87 ± 1.35^a^	60.68 ± 4.29^a^	26.01 ± 1.19^a^
P_2_	84.62 ± 3.70^b^	33.52 ± 1.35^b^	84.62 ± 4.29^b^	33.52 ± 1.19^c^
F_1_	101.66 ± 2.03^c^	34.04 ± 1.35^b^	92.21 ± 4.29^b^	31.66 ± 1.19^bc^
F_2_	88.81 ± 3.70^b^	30.66 ± 0.74^b^	88.80 ± 1.92^b^	32.03 ± 0.53^bc^
BC_1_P_1_	94.70 ± 3.21^bc^	33.06 ± 1.35^b^	83.23 ± 3.03^b^	30.71 ± 0.84^bc^
BC_1_P_2_	96.27 ± 3.70^bc^	31.99 ± 1.17^b^	89.45 ± 5.25^b^	27.88 ± 1.46^ab^

^1^The same letters in the same column indicate no significant differences at the 5% level based on Tukeys Honesty Significant Difference (HSD).

### 3.3 Gene action of iron and zinc

Test for epistasis was done before estimation of genetic variation to help in deciding the method of analysis for the components of variation. The results of simple scaling test for iron and zinc for the two crosses are presented in Table 5. The results showed that in Cal 96 ˣ RWR 2154 cross, scale A and D were significant for iron concentration. While in MCR-ISD-672 ˣ RWR 2154 cross, scale C was significant for iron concentration. For the zinc concentration, scale B was significant while scale D was highly significant in MCR-ISD-672 ˣ RWR 2154 cross. The significant of any of the scales indicated the presence of epistasis.

**Table 5:** Estimates of additive, dominance and epistatic effects (and their standard errors) from the joint scale test for iron and zinc concentration on common beans measured by x-ray fluorescence (XRF) in parents (P_1_ and P_2_) and their F_1_, F_2_, BC_1_P_1_ (F_1_ ˣ P_1_) and BC_1_P_2_ (F_1_ ˣ P_2_) in two crosses grown in CRI research station.

Model	Cross 1		Cross 2	
	Cal 96 ˣ RWR 2154		MCR-ISD-672 ˣ RWR 2154	
	Iron	Zinc	Iron	Zinc
**Scaling test**				
A	26.13 ± 8.62*	6.20 ± 3.32ns	13.58 ± 6.28ns	3.74 ± 2.06ns
B	6.26 ± 7.27ns	−3.58 ± 2.97ns	2.07 ± 9.89ns	−9.41 ± 3.06*
C	5.70 ± 11.61ns	−4.85 ± 4.02ns	25.48 ± 10.03*	5.26 ± 3.14ns
D	−13.35 ± 5.98*	−3.73 ± 1.97ns	4.92 ± 5.75ns	5.46 ± 1.71**
**Joint scaling test**				
M	73.93 ± 1.65***	29.47 ± 0.50***	74.4 ± 1.59***	30.04 ± 0.55***
A	−11.24 ± 1.66***	−3.50 ± 0.50**	−12.48 ± 1.63***	−3.03 ± 0.57*
D	30.76 ± 3.21***	4.20 ± 1.05***	18.85 ± 2.05***	2.16 ± 0.99*
χ2	9.49*	7.09ns	10.35*	17.49***
**Six parameter**				
M	46.42 ± 13.82**	22.23 ± 5.30**	82.48 ± 14.43***	40.7 ± 4.11***
A	−11.51 ± 1.83***	−3.83 ± 0.52***	−11.97 ± 1.75***	−3.76 ± 0.61***
D	114.31 ± 35.74**	21.9 ± 14.09	15.54 ± 38.23	−25.64 ± 11.14*
Aa	26.69 ± 13.69	7.47 ± 5.28	−9.83 ± 14.60	−10.93 ± 4.06*
Ad	19.88 ± 10.55	9.78 ± 4.22*	11.51 ± 11.60	13.16 ± 3.51**
Dd	−59.08 ± 22.94*	−10.08 ± 9.12	−5.81 ± 24.29	16.6 ± 7.28*

*, **, *** indicate terms are significant at P < 0.05, P < 0.01 and P < 0.001, respectively. m = mid-parent effect, a = additive effect, d = dominance effect, aa = additive x additive effect, ad = additive x dominance effect and dd = dominance x dominance effect, ns = non-significant.

The joint scaling test (additive-dominance model) showed the three parameters (m, [a] and [d]) to be significant for all the traits studied (Table 5). This was an indication that additive and dominance genetic effects contributed significantly to the inheritance of seed iron and zinc in both crosses. However, χ^2^ test values (p < 0.05) were significant for iron in the two crosses and zinc in cross MCR-ISD-672 ˣ RWR 2154, indicating that the additive-dominance model was not adequate in explaining the mode of gene action governing high iron and zinc in the crosses. Thus, epistatic interactions were involved in the genetic control of the traits. However, the adequacy of the additive-dominance model for zinc in cross CAL96 ˣ RWR 2154 suggests the absence of non-allelic interaction in the inheritance of the trait. The results showed that both additive and dominance gene effects were implicated in the inheritance of high zinc in cross CAL96 ˣ RWR 2154, indicating ineffective selection for the trait in early segregating generations. Estimates of genetic effects from the six-parameter model indicated that mean and additive effects for both traits in cross CAL96 ˣ RWR 2154 and MCR-ISD-672 ˣ RWR 2154 were significant (Table 5). The significance of the additive gene effect for traits in the crosses suggests the potential for further improvement of iron and zinc through selection from early segregating generations. Dominance [d] and dominance x dominance [dd] were significant in CAL96 ˣ RWR 2154 and MCR-ISD-672 ˣ RWR 2154 for iron and zinc, respectively. Again the estimates of [d] and [dd] had different signs suggesting that the gene interactions were duplicative. Gene interactions are considered to be duplicative when dominance [d] and dominance x dominance [dd] estimates have different signs, confirming the importance of dominance effects [18]. In most cases, the magnitude of dominance [d] gene effects appeared to be higher than additive gene effects for both traits in both crosses. The large positive dominance effect relative to additive effect suggests that the parents are genetically different since dominance is due solely to the heterozygosity of those genes for which the parents differ [26]. Again, the positive and highly significant dominance effect [d] increased iron content in CAL 96 ˣ RWR 2154 cross. The results suggested that iron and zinc contents in common bean may be controlled by both additive and non-additive genes. Similar findings were also reported by Refs. [15,27,28].

### 3.4 Heritability and genetic advance

Heritability (1–8) and genetic advance as percent of mean for iron and zinc concentrations of two common bean crosses are presented in Table 6. Broad-sense heritability (1) estimates were high for iron (62%) and zinc (74%) in Cal 96 ˣ RWR 2154 cross. However, moderate to low narrow-sense of heritability (2) estimates were recorded for both iron (53%) and zinc (21%) in this same cross, respectively. Broad-sense and narrow sense heritability estimates of similar magnitude were observed by Ref. [27] for iron concentrations. Again, their narrow-sense heritability estimates were also similar in magnitude as reported by Ref. [29]. In MCR-ISD-672 ˣ RWR 2154 cross, high to moderate broad-sense heritability estimates for iron (82%) and zinc (60%) were recorded. Again, similar trend was obtained in narrow-sense heritability. High to moderate broad-sense heritability estimates recorded for iron and zinc is an indication of lesser environmental effects on the traits. High to moderate narrow-sense heritability estimates observed for iron in both crosses and zinc in MCR-ISD-672 ˣ RWR 2154 cross indicate that early selection for improvement of the traits would be effective. However, low narrow-sense heritability recorded for zinc in Cal 96 ˣ RWR 2154 cross is an indication that selection for improvement of the trait may not be effective in early segregating generations.

**Table 6:** Variance components, genetic parameter estimates and prediction of gains with selection for iron and zinc concentrations in common beans that were obtained from crosses Cal 96 ˣ RWR 2154 and MCR-ISD-672 ˣ RWR 2154.

**CAL 96 ˣ RWR 2154**										
Trait	P_1_	P_2_	F_1_	F_2_	BC_1_P_1_	BC_1_P_2_	$H^{2}$	$h^{2}$	GA	GAM
Iron	29.60	90.50	71.52	168.09	141.79	104.63	0.62	0.53	16.56	18.71
Zinc	2.86	7.04	8.96	23.97	21.79	21.19	0.74	0.21	7.44	23.77
**MCR-ISD-672 ˣ RWR 2154**										
Iron	20.14	90.50	12.09	233.26	161.56	129.65	0.82	0.75	25.94	30.49
Zinc	6.42	7.04	5.90	16.10	12.96	11.88	0.60	0.46	4.95	15.94

$h^{2}$ = narrow sense heritability, $H^{2}$ = broad sense heritability, GA = genetic advance, GAM = genetic advance as percentage of mean.

A relative comparison of heritability and expected genetic advance will give an idea about the nature of gene action governing a particular trait [30]. Genetic advance as percent of mean (GAM) (10) was moderate to high for iron and zinc in both crosses. The results of this study showed moderate to high broad-sense heritability and GAM for iron and zinc in both crosses, suggesting that the traits are governed by additive genes and selection for improvement of the traits would be rewarding. Heritability combined with genetic advance (9) is a more reliable index for selection of traits [31]; hence, the selection from segregating populations in these crosses would be based on the interpretations given above.

## 4 Conclusion

Genetic variation was observed in crosses CAL 96 ˣ RWR2154 and MCR-ISD-672 ˣ RWR 2154 for iron and zinc content. Additive and non-additive gene effects were both important in the expression high iron and zinc concentrations. Moderate to high broad sense heritability and variable narrow sense heritability were observed for iron and zinc in the two crosses, suggesting that the variation observed among the parents and progenies was largely due to additive gene effects. Thus, selection for improvement of iron and zinc in early segregating generations would be effective. The results of the present study generally showed iron and zinc superiority of the F_1_ hybrids over the mid- and better parent in common bean.

## Declarations

### Author contribution statement

Maxwell Lamptey: Conceived and designed the experiments, Performed the experiments and Wrote this paper.

Richard Adu Amoah: Analyzed and interpreted the data and Contributed reagents, materials, analysis tools or data.

Francis Osei Amoako-Andoh, Louis Butare, Kwabena Asare Bediako, Isaac Tawiah and Stephen Yeboah: Analyzed and interpreted the data and Contributed reagents, materials, analysis tools or data.

James Yaw Asibuo and Hans Adu-Dapaah: Conceived and designed the experiments and Wrote the paper.

### Data availability statement

Data will be made available on request.

### Additional information

No additional information is available for this paper.

## Declaration of competing interest

The authors declare that they have no known competing financial interests or personal relationships that could have appeared to influence the work reported in this paper.
